# Endoscopic Identification of a Hidden Internal Opening Using Video-Assisted Anal Fistula Treatment (VAAFT)

**DOI:** 10.7759/cureus.92821

**Published:** 2025-09-21

**Authors:** Yasemin Yildirim, Onur Bayraktar, Ilknur Erenler Bayraktar

**Affiliations:** 1 General and Colorectal Surgery, Florence Nightingale Hospital, Istanbul, TUR; 2 General and Colorectal Surgery, Liv Hospital Vadistanbul, Istanbul, TUR; 3 General and Colorectal Surgery, Private Practice, Istanbul, TUR

**Keywords:** anal fistula, complex fistulas, endoscopic treatment, recurrence, video-assisted anal fistula treatment (vaaft)

## Abstract

The purpose of this study is to present our experience with video-assisted anal fistula treatment (VAAFT) in a patient with a complex and recurrent anorectal fistula, whose internal opening could not be identified through multiple prior surgical interventions.

A 36-year-old male patient with a history of nine previous fistula surgeries presented with persistent symptoms and a newly developed lateral external opening in the right gluteal region. VAAFT was performed under general anesthesia with the patient in the prone position. The fistuloscope was advanced through the external opening, allowing visualization of both the primary and secondary tracts. The internal opening was identified, and the fistulous tracts were thoroughly debrided. A draining seton was placed while preserving the integrity of the sphincter complex.

At the four-month follow-up, all secondary tracts had successfully closed. However, due to the presence of false tracts resulting from prior interventions, the primary tract had evolved into a suprasphincteric fistula. The patient remains under regular follow-up with a seton in place and has reported significant improvement in both symptoms and overall quality of life.

Our experience indicates that VAAFT is a valuable option in selected cases of complex and recurrent anal fistulas, especially when the internal opening cannot be identified using conventional methods.

## Introduction

In the anal canal, approximately 8 to 10 crypt glands are circumferentially arranged at the level of the dentate line [1]. These glands extend through the internal sphincter and terminate within the intersphincteric plane [2]. In over 90% of cases, anorectal fistulas originate from infections of these cryptoglandular structures [3]. An anorectal fistula is defined as an abnormal connection between two epithelialized surfaces, most commonly linking an infected anal gland abscess to the perianal skin, and occasionally to adjacent pelvic organs. Other etiological factors include Crohn’s disease, obstetric trauma, radiation proctitis, foreign bodies, infectious processes, and malignancies.

The choice of treatment depends on several factors, including the underlying etiology, anatomical location, type, and chronicity of the fistula. Accurate localization of the internal opening is critical for successful surgical management, as failure to do so is a major cause of recurrence. In complex or recurrent cases, conventional techniques, such as probing, dye injection, or examination under anesthesia, may fail to detect the internal opening due to anatomical distortion, fibrosis, or the presence of false tracts created by previous interventions.

Minimally invasive techniques have gained increasing popularity in the treatment of anal fistulas. Among these, video-assisted anal fistula treatment (VAAFT) has emerged as a promising approach. VAAFT is an endoscopic, sphincter-preserving procedure that allows the direct visualization of the entire fistula tract and internal opening using a fistuloscope [4].

We report a challenging case of a complex anorectal fistula in a patient with a history of nine prior unsuccessful surgical interventions, where the internal opening had remained undetected. In this case, the internal opening was successfully identified using the VAAFT technique.

## Case presentation

A 36-year-old male patient presented with persistent discharge, pruritus, and pain. The patient had experienced multiple episodes of recurrent perianal abscesses, which were managed with incision and drainage. Over the past year, he developed continuous purulent discharge from a newly formed external opening in the right gluteal region, lateral to the ischial tuberosity. His symptoms progressively worsened, leading to a marked decline in quality of life. MRI revealed a complex fistula with more than three secondary tracts, the longest of which extended approximately 20 cm and opened into the right gluteal region. The primary tract was located at the 7-8 o’clock position in relation to the rectum.

VAAFT was undertaken with the primary aim of accurately localizing the internal opening and comprehensively mapping the fistulous tract architecture. The procedure was conducted under general anesthesia with the patient positioned in the prone jackknife posture to optimize perineal access. Following standard antiseptic preparation, the fistuloscope was carefully introduced through the external opening, allowing real-time endoluminal visualization of the primary tract and any secondary extensions. Continuous irrigation facilitated clear visualization of the epithelialized tract; the internal opening was clearly identified and confirmed by both visual inspection and concurrent transillumination. Thorough curettage and unroofing of the secondary tracts were performed to disrupt the fistulous epithelium and promote healing. A loose draining seton was inserted to maintain adequate drainage and prevent premature closure, thereby minimizing the risk of abscess formation or recurrence. Importantly, the procedure ensured complete preservation of the sphincter mechanism. All surgical steps are demonstrated in Video 1.

**Video 1:** Video-assisted anal fistula treatment

At the four-month follow-up, all secondary tracts had successfully closed. However, due to the development of false tracts from prior surgical attempts, the primary tract had progressed to a suprasphincteric course. Consequently, only the main fistulous tract remains, and the patient continues to be followed with a draining seton in place.

## Discussion

The management of complex anorectal fistulas remains a formidable challenge in colorectal surgery, primarily due to the difficulty in accurately identifying the internal opening and the need to balance effective treatment with sphincter preservation. Failure to detect the internal opening is a leading cause of recurrence, especially in patients with a history of multiple unsuccessful procedures, as seen in the present case.

In this patient, nine prior interventions had failed to identify the internal opening, underscoring the limitations of traditional surgical approaches in the context of altered anatomy and chronic infection. Repeated incision and drainage, although temporarily alleviating abscess formation, may contribute to the formation of secondary and false tracts, further complicating subsequent surgical exploration. This highlights the need for advanced diagnostic and therapeutic modalities capable of providing direct visualization and precise anatomical mapping.

VAAFT, first described by Meinero et al. in 2011, has emerged as a minimally invasive and sphincter-sparing technique that allows direct endoscopic access to the fistula tract [5]. The use of a fistuloscope facilitates real-time visualization of the entire tract system, identification of secondary extensions, and crucially, localization of the internal opening via direct inspection and transillumination. A meta-analysis published in 2023 reported that VAAFT allowed for the identification of the internal opening in approximately 98% of cases [6]. In the present case, the internal opening was successfully identified for the first time using VAAFT, despite previous failures with conventional techniques.

Healing rates exceeding 80% have been reported with VAAFT [7]. Recurrence rates within the first year have been reported to range between 17% and 24%. While these outcomes suggest favorable healing, current evidence remains limited, and further high-quality studies are needed to establish definitive conclusions. The main advantage of VAAFT lies in its high success in identifying the internal orifice, which is critical for effective treatment of complex anal fistulas. There are comparative studies in the literature evaluating the efficacy of VAAFT. In a study comparing VAAFT with ligation of the intersphincteric fistula tract (LIFT), similar outcomes were observed in terms of days to healing, failure rates, and recurrence rates [8]. In addition, a meta-analysis published in 2025 reported that VAAFT achieved higher healing rates compared to LIFT and fistula laser closure (FiLaC) [9]. While sphincter-related complications were observed in patients undergoing LIFT, no such adverse effects were reported in those treated with VAAFT. Moreover, the Italian Society of Colorectal Surgery currently recommends VAAFT with a level of evidence 2 [10]. Therefore, further research involving larger patient cohorts and longer follow-up periods is necessary to generate more robust and conclusive evidence regarding the clinical utility.

To date, there are no dedicated studies assessing the cost-effectiveness or learning curve of the VAAFT procedure, and technical literature remains limited. Nevertheless, the method is feasible in centers equipped with standard laparoscopic systems, requiring only the acquisition of a fistuloscope. For surgeons with laparoscopic experience, the technique is reproducible and technically accessible.

## Conclusions

In this challenging case, VAAFT enabled precise localization of the internal opening and detailed mapping of the complex fistula network. Video-assisted techniques may be a valuable tool in the management of recurrent and anatomically complex anal fistulas. Further studies with larger patient populations and longer follow-up are needed to better define the role and long-term efficacy of VAAFT.
